# Correction: Cortex commands the performance of skilled movement

**DOI:** 10.7554/eLife.22214

**Published:** 2016-10-20

**Authors:** Jian-Zhong Guo, Austin R Graves, Wendy W Guo, Jihong Zheng, Allen Lee, Juan Rodríguez-González, Nuo Li, John J Macklin, James W Phillips, Brett D Mensh, Kristin M Branson, Adam W Hantman

Guo JZ, Graves AR, Guo WW, Zheng J, Lee A, Rodríguez-González J, Li N, Macklin JJ, Phillips JW, Mensh BD, Branson K, Hantman AW. 2015. Cortex commands the performance of skilled movement. *eLife* **4**:e10774. doi: 10.7554/eLife.10774.

Published 2, December 2015

In the published article one of the references cited the wrong article.

In the Discussion where Wang et al., 2010 was cited, it should have cited Wang et al., 2011.

The correct citation is shown below:

Wang L, Conner JM, Rickert J, Tuszynski MH. 2011. Structural plasticity within highly specific neuronal populations identifies a unique parcellation of motor learning in the adult brain. Proceedings of the National Academy of Sciences of USA 108:2545-2550. doi: 10.1073/pnas.1014335108

The originally published citation is also shown for reference:

Wang Z, Kraft JJ, Hui AY, Miller WA. 2010. Structural plasticity of barley yellow dwarf virus-like cap-independent translation elements in four genera of plant viral RNAs. Virology 402:177–186. doi: 10.1016/j.virol.2010.03.025

The article has been corrected accordingly.

